# Supplementary material for: A continuum of bright and dark-pulse states in a photonic-crystal resonator
Source: Nat Commun. 2022 Jun 6;13:3134. doi: 10.1038/s41467-022-30774-x (PMC9170700; doi:10.1038/s41467-022-30774-x)
Supplement: Supplementary file 1 — Supplementary Information [file 41467_2022_30774_MOESM1_ESM.pdf]

Supplementary Information for: A continuum of bright- and dark-pulse states in photonic-crystal resonators

Su-Peng Yu[1,2], Erwan Lucas[1,2], Jizhao Zang[1,2], and Scott B. Papp[1,2,*]
[1]Time and Frequency Division, National Institute of Standards and Technology, Boulder, CO 80305, USA
[2]Department of Physics, University of Colorado, Boulder, CO 80309, USA
[*]Correspondence email address: scott.papp@nist.gov

I. PHASE-MATCHING IN NORMAL DISPERSION

Phase-matching for pattern generation is conventionally unavailable in normal dispersion resonators. In this work, this is overcome using the photonic crystal shift. Figure S1**a** illustrates the integrated dispersion $D_{\text{int}}(\mu) = \omega_\mu - \omega_0 - \mu D_1$, where ω_μ stands for the cold-cavity frequencies for the μ-th mode from the pump mode $\mu = 0$, ω_0 the pump mode frequency, and D_1 the local mode spacing or free-spectral range. As the $\mu = 0$ mode is energized by a pump laser, optical intensity builds up in the resonator, causing the modes to shift toward lower frequencies under the Kerr effect. However, the pump mode self-frequency shift $\delta_{\mu=0} = \frac{1}{2}\delta\mu' \neq 0$ is a factor of two smaller than the other modes experiencing cross phase modulation [38]. In an anomalous dispersion system, the D_{int} curve has positive curvature, meaning the frequency difference from the local mode spacing increases with μ'. This compensates for the Kerr shift mismatch between the $\mu = 0$ mode and the $\mu' \neq 0$ modes, therefore enabling four-wave mixing (FWM) phase-matching. In the normal dispersion case shown in Fig. S1**a**, the curvature is negative, moving the $\mu' \neq 0$ modes further away from FWM phase-matching. Therefore, Kerr comb generation is absent in the conventional normal dispersion resonators.

A point-defect at the pump mode ϵ_{PhC} re-enables the FWM matching, shown in Fig. S1**b**, by filling in the mismatch between a desired pair of modes $\pm\mu'$ and the $\mu = 0$ mode. The FWM matched modes can energize to form Turing patterns similar to the anomalous dispersion case. More importantly, the pump mode shift modifies the detuning ranges where the pulse patterns form [25], also visible in main text Eq. (1). The stationary waveforms in the Kerr resonator like the bright- or dark-pulse states compose of many interlocking modes. Their component mode frequencies pull into alignment with each other by Kerr shift [39], shown in Fig. S1**c**. Since the self- and cross-phase modulation difference does not depend on the sign of dispersion, the counter-balancing term in Eq. (1) can also be interpreted as the time-domain equivalent of the Kerr-mismatch balancing in Ref. [25].

II. DERIVATION OF LOCAL ENERGY FLOW

We provide the derivation for Main Text Eq.(2) by evaluating the rate of change for the intensity $I(\theta)$, which is the energy per unit θ. We calculate this quantity by substituting Main Text Eq. (1) into the expression $\partial_t I(\theta)$:

$$\begin{aligned}\partial_t I =& \psi^* \partial_t \psi + h.c. \\ =& -2|\psi|^2 - \frac{i\beta}{2}(\psi^* \partial_\theta^2 \psi - \psi \partial_\theta^2 \psi^*) \\ &+ F(\psi^* + \psi) + i\epsilon(\psi^* \bar{\psi} - \psi \bar{\psi}^*) \\ =& -2I + \frac{\beta}{2} \cdot 2\partial_\theta \cdot \mathbb{I}m(\psi^* \partial_\theta \psi) \\ &+ 2F \cdot \mathbb{R}e(\psi) + 2\epsilon \cdot \mathbb{I}m(\psi \bar{\psi}^*) \\ =& 2\Big(- \rho_{\text{loss}} - \nabla_\theta \cdot J_\beta + \rho_{\text{in}} + P_{\text{PhC}} \Big)\end{aligned}$$

where ρ_{in}, ρ_{loss} are the energy in-flow and loss defined in the Main Text, $J_\beta = -\frac{\beta}{2} \cdot \mathbb{I}m(\psi^* \partial_\theta \psi)$ is an energy current driven by dispersion, and $P_{\text{PhC}} = \epsilon \cdot \mathbb{I}m(\psi \bar{\psi}^*)$ represents power exchange induced by the photonic shift ϵ. We identify ρ_{in} as the energy flow of the physical pump as it is proportional to F. For the discussion of the two intensity levels, the local field is approximately flat, $J_\beta \simeq 0$. We also note that P_{PhC} exchanges energy within the resonator, but $\oint P_{\text{PhC}} \cdot d\theta = \mathbb{I}m(|\bar{\psi}|^2) = 0$, conserving the total energy.

III. BANDWIDTH TO FILLING-FRACTION CORRESPONDENCE

We derive the inverse relation between fitted bandwidth BW in main text Fig. 4 and the filling fraction parameter t_c in the theoretical analysis. We begin with the square-wave function:

$$\psi(\theta) = \Theta(\pi t_c - |\theta|)$$

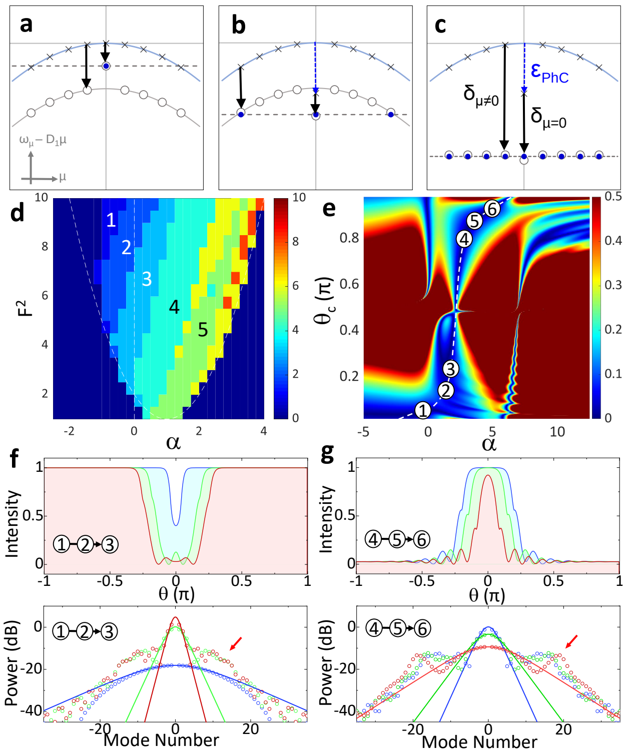

Figure 1. Spectral-temporal features: Kerr shift δ_μ influencing mode frequency ω_μ relative to a constant grid $D_1\mu$ for **a** a normal dispersion base ring, **b** the Turing pattern and **c** the pulse state in the PhCR with shift $\epsilon_{\rm PhC}$, showing the cold-cavity ($\times$) and Kerr-shifted ($\circ$) frequencies, and energy in some modes ($\bullet$). **d** Diagram showing number of fringes in the optical waveform. **e** Error measure of the analytical Ansatz using Eq. S4 (for $F = 3.0$), where a low-error valley (dashed white line) indicates reducing filling fraction with detuning. **f-g** show the intensities and spectra of the Ansatz along the curve in **e**, sweeping from **f** dark soliton toward half-filled, and **g** half-filled toward bright pulse. The center lobe fits on the spectra (plain lines) are $y = 10\log_{10}(\text{sech}^2(\frac{x-x_0}{BW}))$.

where $\Theta(\theta)$ is the Heaviside function. We obtain the modal spectrum of this waveform by carrying out the Fourier transform:

$$v(m) = \hat{\mathcal{F}}(\psi) = \frac{\sin(m\pi t_c)}{m\pi}$$

where we note that setting $t_c \to 1 - t_c$ changes the modal phase by $(-1)^{m+1}$ but leaves the magnitude invariant, as a result of the bright- and dark-pulse correspondence.

We focus on the center-lobe near $m = 0$ and calculate the second-order derivative which links to the bandwidth. Specifically, we compute the second-order derivative for the spectral power in units of dB, $S(f) = 10\log_{10}(|v(f)|^2)$, where the mode frequencies are $f = c + m \cdot \text{FSR}$, where FSR is the free-spectral range. Evaluating the second derivative of S at $m = 0$, we get:

$$\partial_f^2 S(f = c) = -\frac{20}{\ln(10)} \frac{\pi^2 t_c^2}{3\text{FSR}^2}$$

where $\ln(x)$ is the natural-log function. We compare this to the fitting function to extract BW:

$$y = a + 10\log_{10}\left[\text{sech}^2((f-c)/\text{BW})\right]$$

by taking its second-order derivative at $f = c$, we get:

$$\partial_f^2 y(f = c) = -\frac{20}{\ln(10)} \cdot \frac{1}{\text{BW}^2}$$

Comparing the two forms, we obtain the relation between BW and t_c:

$$\frac{1}{\text{BW}^2} = \frac{\pi^2 t_c^2}{3\text{FSR}^2}$$

taking the square-root of both sides, we get the relation:

$$\text{BW} = \frac{\sqrt{3}\text{FSR}}{\pi} \cdot t_c^{-1}$$

which shows the inverse-proportional relation between BW and t_c, or, in the case of the dark-pulse-like ranges:

$$\text{BW} = \frac{\sqrt{3}\text{FSR}}{\pi} \cdot (1 - t_c)^{-1}$$

In summary, the spectral manifestation of varying temporal filling fraction with laser detuning is an inverse-proportional change in the center-lobe bandwidth.

IV. WAVEFORM FEATURES

The waveforms in normal dispersion systems show several distinct features. The main text focuses on the center lobe and its implication on the temporal duration of the bright- or dark-pulse. There are additional features at higher azimuthal frequencies including the 'wing' and 'horn'. The wing feature arises from the dark pulse touching down to zero-intensity and developing internal features (red arrow in Fig. S1**f**). The horn feature comes

from the oscillating patterns near the body of the bright-pulse (red arrow in Fig. S1**g**). The phase diagram of the developing of these patterns, shown by plotting the number of local minima in the field, is shown in Fig. S1**d**. We demonstrate the origin of these features using a locally linearised LLE with a piecewise-constant intensity nonlinear term. This allows us to derive an approximate waveform solution for given system parameters (detuning α and angle θ_c separating the two piecewise domains). The panels Fig. S1**f**,**g** are created using this method and reasonably approximate the full LLE waveforms.

We write the LLE in the following form:

$$\partial_\tau \psi = -(1+i\alpha)\psi - \frac{id_2}{2}\partial_\theta^2\psi + iI(\theta)\psi + F \quad (1)$$

where $I(\theta)$ is the local intensity at azimuthal angle θ. We approximate the equation by separating the resonator into the domains $|\theta| < \theta_c$ and $|\theta| > \theta_c$, where θ_c specifies a switching azimuthal angle between the two domains, related to the filling ratio by $\theta_c = (1-t_c)\,\pi$. We then assume $I(\theta)$ can be treated at a constant for each domain, switching between two fixed levels I_j, $j = 1, 2$. This method is reminiscent of the switching waves [31], but is subjected to the edge-less boundary conditions of the ring resonator. The particular solution to the system are constant fields sourced by the pump field F that produce the intensities I_j in a self-consistent manner. We then search for the general solutions to Eq. S1 under this approximation. The linearized second-order differential equation in θ reads:

$$\frac{i\beta}{2}\partial_\theta^2\psi = -(1+i\alpha_j)\psi \quad (2)$$

where $\alpha_j = \alpha - I_j$, $j = 1, 2$ are constants for each domain. The equation yields general solutions of the form $\exp(\lambda_\pm\theta)$, where $\lambda_\pm = \pm\frac{1}{\sqrt{\beta}}(u(\alpha_j) + iu(-\alpha_j))$, $u(x) = \sqrt{\sqrt{x^2+1} - x}$. We choose solution functions with the form:

$$\psi_j(\theta) = A_j\cosh(\lambda_j\theta) + E_j \quad (3)$$

for each domain, where E_j is the background field for level j, $I_j = |E_j|^2$, and the hyperbolic cosine function is selected to respect the symmetry in $\pm\theta$. The pattern of the complex hyperbolic cosine can be exponential-like or sine-like depending on the signs of the local α_j. Finally, we set the the background level E_1 for $|\theta| < \theta_c$, and E_2 for $|\theta| > \theta_c$. We solve for the coefficients A_j by requiring field continuity $\psi_1(\theta_c) = \psi_2(\theta_c)$, and continuity of the derivatives $\psi_1'(\theta_c) = \psi_2'(\theta_c)$. This process provides us with an analytical Ansatz for the field in the resonator, for each parameter set $(\beta, \alpha, \theta_c, E_j)$. We find this simple approximate solution is sufficient to reproduce the spectral features we observed.

We identify the Ansatz parameters best matching the physical state by minimizing their error in the time-stationary LLE. In particular, we search for a fitness measure that is sensitive to the intensity filling fraction by examining the role of each mode order μ. The pump mode $\mu = 0$ creates a flat background, which interferes with mode $\mu' = \pm 1$ to create a simple cosine modulation. At this point, the filling fraction is exactly 0.5 due to the shape of the cosine function. Adding $\mu' = \pm 2$ terms, we begin to modify the filling ratio, depending on the relative phase between the modes. For example, $1 + 0.75\ cos(\theta) + 0.25\ cos(2\theta)$ creates a bright pulse, while $1 + 0.75\ cos(\theta) - 0.25\ cos(2\theta)$ creates a dark pulse. With this in mind, we create a fitness function depend on the $|\mu| \leq 2$ modes. We create a simple form by dividing the modal equations for mode 1 and 2 by their respective field amplitudes, and subtracting the two. This creates an advantageous form that eliminates explicit dependence on the pump mode shift ϵ_{PhC} or the pump field parameters α, F:

$$\xi_{12} = |\,(\delta_2 - d_{\mathrm{int}}(2)) - (\delta_1 - d_{\mathrm{int}}(1))\,| \quad (4)$$

where δ_j, j=1,2 are the modal Kerr shift [25]:

$$\delta_\mu = \mathrm{Re}\left(\hat{\mathcal{F}}\{|\psi(\theta)|^2\psi(\theta)\},_\mu / \hat{\mathcal{F}}\{\psi(\theta)\},_\mu\right) \quad (5)$$

and d_{int} are the linewidth-normalized integrated dispersion. The ξ_{12} term vanishes for time-stationary solutions of the LLE. We use this metric to minimize the error of our Ansatz. Figure S1**e** shows the evolution of ξ_{12} as a function of (α, θ_c), where we have assumed the two intensity levels $I_{1,2}$ correspond to the upper- and lower-state of the bi-stable pump mode on resonance [19] for $F = 3.0$. ξ_{12} shows three minimum valleys. The center valley (dashed line in Fig. S1**e**) shows the correct exponential-like shape for the upper level and sine-like shape for the lower, in agreement to calculated intensity patterns in the LLE. Tracing this minimum valley of ξ_{12}, we observe the trend of increasing α resulting in decreasing t_c (increasing θ_c). This suggests that the Ansatz functions, while crude, seem to capture important aspects of the system. The intensity patterns and spectra along the curve are shown in Figure S1**f**,**g**, at point 1–3 for the dark pulse, and 4–6 for the bright pulse.

The Ansatz functions show the shared physical origin of the lobe-number inside a dark-pulse (Fig. S1**f**) and oscillations on the sides of the bright-pulse (Fig. S1**f**). These two appear spectrally as the ‘wing’ and ‘horn’ features,

respectively. The oscillations result from the sine-like behaviour of the linearized LLE in the low-intensity domain, while the periodicity approximately scales with $\sqrt{\beta}$ from the form of the eigenvalue $\lambda_{\pm}$. The upper level of the pulse has exponential-like waveform, therefore does not show the oscillations.

V. EXISTENCE RANGE AND AVAILABLE COMB STATES

The ranges of comb states available in a PhCR depends on the mode-shift ϵ. Varying ϵ enabled us to access different ranges of the continuum, such as Main Text Fig. 4**c** and **d** showing the bright- and dark-pulse sides of the continuum. In addition, Figure S2**a** shows accumulated optical spectra measured consecutively over an α sweep across the middle-section of the continuum and the center-lobe tuning reversal behavior. We see the center lobe bandwidth (proportional to μ) first decreases with α, reaches a minimum, then increases with α. This data set shows an extended section of the continuum, but the reachable states on the bright end of the continuum are limited by the existence range. The blue dot on the lower-right-most corner of Fig. S2**a** results from the comb state being lost before the last spectrum data was completed, indicating that α has tuned out of the comb existence range for the device. This limited range of α and available range of states makes accessing the full continuum in a single device experimentally challenging.

We explore the existence range and available comb states using the LLE. The comb power traces are shown in Fig. S2**b**. The three traces are calculated using the same parameter set as Main Text Fig. 1**b** but with ϵ= 2, 4, and 8. While the pump power is identical for the three traces, the ranges where comb power is nonvanishing is different. For example, the ϵ= 2 case loses its comb state at higher α than the other two cases. The finite comb existence range limits the states available to a given device. The ϵ= 2 case (which is identical to Main Text Fig. 1**b**) lives in a small parameter space range where the full continuum can be addressed. This is shown in Figure S2**c** and **d** where the minimum-detuning state reachable for ϵ= 2 yields a single-lobe dark soliton, while the maximum-detuning state is a partially-filled state approaching the platicon state. At ϵ= 4, the low-detuning side of the sweep can only reach a partially-filled state, while at ϵ= 8 the comb remains in the platicon state throughout the sweep. We therefore assemble the full picture of the continuum by varying ϵ over a set of otherwise identical devices. By combining the three cases in Fig. S2, we cover a wider stretch of the continuum than available to any individual device, from the single-lobe dark-soliton to the deep platicon state with short duration and enhanced intensity.

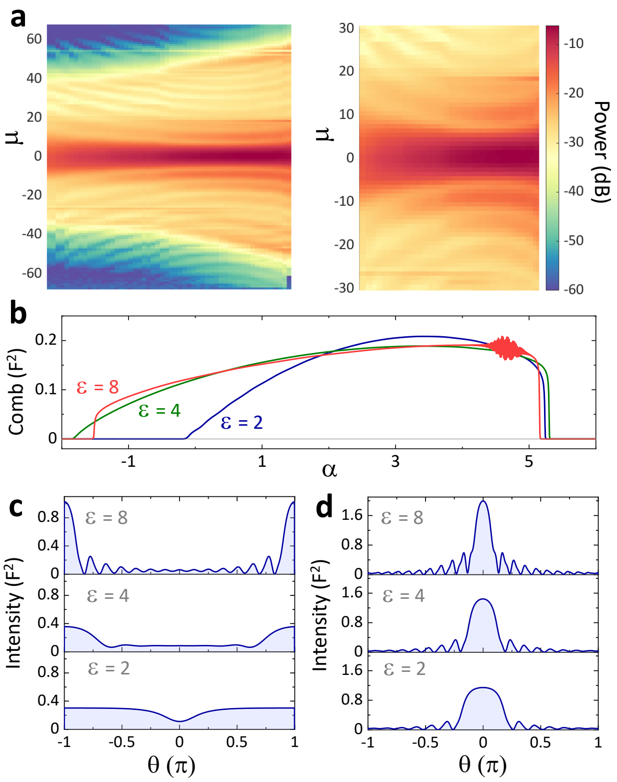

Figure 2. Tuning across the continuum: **a** Accumulated optical spectra of an experimental α sweep showing the center-lobe tuning reversal. A zoom-in plot of the center-lobe is shown to the right. **b** Simulated comb power traces with PhCR shift $\epsilon = 2, 4, 8$ and their **c** lowest-detuning and **d** highest-detuning reachable states.

VI. SAMPLE DETUNING SWEEP TRACES

We provide sample oscilloscope traces at low and high power during detuning sweeps from low to high α (high to low laser frequency) in Fig.S 3. We first perform α sweep at low power (Fig.S 3**a**). We observe the split mode of the PhCR as dips in device transmission. Due to residual pump laser reflection at the facets of the chip, we also observe fringes with low contrast that results in the sinusoidal shape of the transmission trace when off-resonance to the PhCR. The PhCR reflects fraction of the pump laser when on-resonance, which interferes with the residual facet reflection to create the reflection lineshape

in Fig.S 3**a**. We extract the passive properties including linewidth and PhC splitting using the low-power laser sweep traces.

Figure S 3**b** shows the same device at higher pump laser power. The high pump can overcome the comb threshold power, but causes significant thermal shift. We observe the transmission showing the typical 'thermal triangle' shape. We note that here only the red-shifted mode of the PhCR split mode is excited. This is achieved by careful control of the starting α for the sweep so it begins in between the two modes. We observe the spontaneous comb power onset as the dark soliton forms. In some cases with larger PhCR splitting, the system can begin in Turing patterns and then transition to the dark soliton state, leading to more complex comb power traces than the case shown here. Note the comb power shows significant detuning dependence, in contrast to the step-like behavior of the anomalous GVD Kerr solitons.

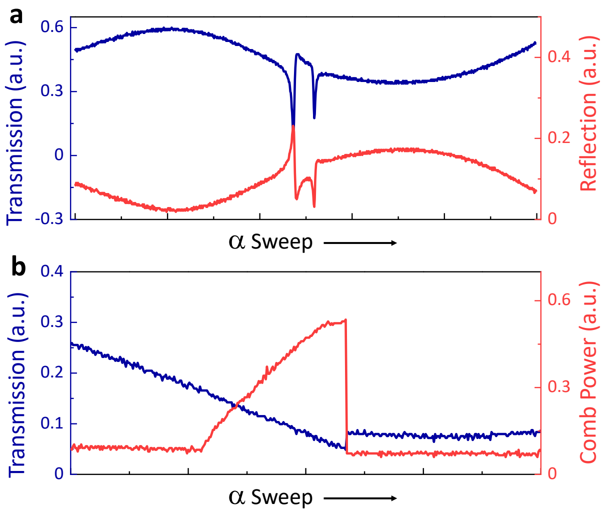

Figure 3. Sample sweep traces: Detuning sweep traces taken at **a** low pump power showing pump transmission and reflection, and **b** at typical power level for comb generation.

VII. POWER CONVERSION EFFICIENCY

The 25% theoretical limitation to power conversion efficiency results from an interplay between pump mode coupling condition and energy distribution of the Kerr effect. The Kerr effect extract energy from the pump mode and distributes it to the comb modes, resulting in an additional loss term on the $\mu = 0$ mode, corresponding to the imaginary counterpart of Eq. S5. The pump mode coupling condition is affected by the Kerr term, in the form of a modified effective loss rate $\kappa_i^{\text{pump}} = \kappa_i + \kappa_{\text{Kerr}}$, where κ_i, κ_{Kerr} stand for the intrinsic and Kerr-induced loss in the resonator. In the critical or near-critically coupled devices in this work, the comb formation becomes a self-limiting process. Increasing the pump power, thus increasing the strength of the Kerr-induced loss term, shifts the coupling condition toward under-coupling, preventing the pump power from entering the resonator. We study the optimal power-conversion pump F^2 in the LLE to find $\kappa_i^{\text{pump}} = \kappa_i + \kappa_c$ at the case giving the 25% efficiency, an analogue condition to maximizing the in-cavity intensity in a Fabry-Perot resonator.

The total power efficiency $\eta_{\text{total}} = P_{\text{comb}}^{\text{out}}/P_{\text{pump}}^{\text{in}}$ can be improved by overcoupling the resonator. We specify the coupling condition by defining the coupling constant $K = \kappa_c/\kappa_i$. The total power efficiency can be related to the internal efficiency η in the form:

$$\eta_{\text{total}} = \frac{4K}{K + K^{-1} + 2} \cdot \eta = \left(\frac{2K}{K+1}\right)^2 \cdot \eta \qquad (6)$$

where $K > 1$ indicates over-coupling. The improved efficiency is achieved at the cost of increased threshold power, therefore reducing the maximum F^2 values that can be achieved by a given pump laser. The benefit of overcoupling arrives from that the absorption loss of the resonator is diluted by the rapid removal of energy by the bus waveguide, thus enabling us to overcome the 25% energy division limit. Note that in the limit of high coupling $K \gg 1$, $\eta_{\text{total}} \to 4\eta$, therefore the maximum internal efficiency of 25% corresponds to an 100% total efficiency in the limiting case. We are actively exploring stronger coupling parameter spaces, and also methods to control the pump mode coupling strength deferentially from the other modes, such as modified bus waveguides with a low-finesse pump recycling cavity [40] or contra-directional grating coupler [41], to further improve the conversion efficiency.

VIII. TABLE OF DEVICE PARAMETERS

Table S1: Device parameters.

Data Set	Ring Width	Ring Radius (from center)	PhC Period	PhC Amplitude	Linewidth (FWHM)	Splitting
Fig. 1c (i)	1.9 um	45.0 um	660	6 nm	350 MHz	1.34 GHz
Fig. 1c (ii)	2.2 um	112.5 um	1662	8 nm	237 MHz	1.25 GHz
Fig. 1c (iii)	2.2 um	112.5 um	1672	8 nm	110 MHz	1.13 GHz
Fig. 4b	2.25 um	112.5 um	1662	13 nm	339 MHz	2.04 GHz
Fig. 4c	1.9 um	45.0 um	664	8 nm	368 MHz	1.66 GHz
Fig. 4d	1.9 um	45.0 um	664	12 nm	473 MHz	2.80 GHz
Fig. 5a	2.25 um	109.5 um	1618	8 nm	110 MHz	1.11 GHz
Fig. 5c	2.25 um	112.5 um	1662	17 nm	375 MHz	2.77 GHz
Fig. 5e	2.0 um	112.5 um	1662	11 nm	231 MHz	2.39 GHz

REFERENCES

[38] T. Herr, M. L. Gorodetsky, and T. J. Kippenberg, “Dissipative kerr solitons in optical microresonators,” in *Nonlinear Optical Cavity Dynamics: From Microresonators to Fiber Lasers*, ch. 6, Wiley-VCH Verlag GmbH & Co, 2015.

[39] C. Bao and C. Yang, “Mode-pulling and phase-matching in broadband kerr frequency comb generation,” *Journal of the Optical Society of America B*, vol. 31, pp. 3074–3080, 2014.

[40] S. Sato, M. Ohashi, M.-K. Fujimoto, M. Fukushima, K. Waseda, S. Miyoki, N. Mavalvala, and H. Yamamoto, “High-gain power recycling of a fabry–perot michelson interferometer for a gravitational-wave antenna,” *Applied Optics*, vol. 39, pp. 4616–4620, 2000.

[41] W. Shi, X. Wang, C. Lin, H. Yun, Y. Liu, T. Baehr-Jones, M. Hochberg, N. A. F. Jaeger, and L. Chrostowski, “Silicon photonic grating-assisted contra-directional couplers,” *Optics Express*, vol. 21, pp. 3633–3650, 2013.
